# Two major quantitative trait loci controlling the number of seminal roots in maize co-map with the root developmental genes *rtcs* and *rum1*

**DOI:** 10.1093/jxb/erw011

**Published:** 2016-02-13

**Authors:** Silvio Salvi, Silvia Giuliani, Claudia Ricciolini, Nicola Carraro, Marco Maccaferri, Thomas Presterl, Milena Ouzunova, Roberto Tuberosa

**Affiliations:** ^1^DipSA University of Bologna, viale Fanin 44, 40127 Bologna, Italy; ^2^KWS SAAT SE, Grimsehlstr. 31, D-37555 Einbeck, Germany

**Keywords:** Maize, primary root, QTL, root architecture, seminal roots, stress tolerance.

## Abstract

The genetic dissection of root architecture and functions allows for a more effective and informed design of novel root ideotypes and paves the way to evaluate their effects on crop resilience to a number of abiotic stresses. In maize, limited attention has been devoted to the genetic analysis of root architecture diversity at the early stage. The difference in embryonic (including seminal and primary) root architecture between the maize reference line B73 (which mostly develops three seminal roots) and the landrace Gaspé Flint (with virtually no seminal roots) was genetically dissected using a collection of introgression lines grown in paper rolls and pots. Quantitative trait locus (QTL) analysis identified three QTLs controlling seminal root number (SRN) on chromosome bins 1.02, 3.07, and 8.04–8.05, which collectively explained 66% of the phenotypic variation. In all three cases, Gaspé Flint contributed the allele for lower SRN. Primary root dry weight was negatively correlated with SRN (*r*= −0.52), and QTLs for primary root size co-mapped with SRN QTLs, suggesting a pleiotropic effect of SRN QTLs on the primary root, most probably caused by competition for seed resources. Interestingly, two out of three SRN QTLs co-mapped with the only two known maize genes (*rtcs* and *rum1*) affecting the number of seminal roots. The strong additive effect of the three QTLs and the development of near isogenic lines for each QTL in the elite B73 background provide unique opportunities to characterize functionally the genes involved in root development and to evaluate how root architecture affects seedling establishment, early development, and eventually yield in maize.

## Highlight

The analysis of a maize elite×landrace introgression library enabled the mapping of major QTLs for seminal root architecture, two of which co-map with known root developmental genes.

Received 21 October 2015; Revised 4 January 2016; Accepted 7 January 2016

## Introduction

Roots are highly specialized plant organs playing a vital role in plant growth and adaptation. Advances in root genetics and physiology provide the opportunity for breeding programs to design and introduce novel root architectural and physiological ideotypes optimized for improved crop adaptation to a range of environmental stresses and increased sustainability of cropping systems (De Dorlodot *et al.*, 2007; Hammer *et al.*, 2009; Tardieu and Tuberosa, 2010; Lynch, 2013; White *et al.*, 2013; Meister *et al.*, 2014).

The maize root apparatus comprises the embryonic and the post-embryonic systems, the latter including all shoot-borne roots that are formed at the consecutive shoot nodes as well as all lateral roots developed from them (Hochholdinger, 2009). The embryonic roots include primary and seminal roots. The primary root, or radicle, differentiates at the basal end of the embryo and is the first root to emerge from the seed at germination. In the embryo, seminal roots are laid down between 22 d and 40 d after pollination and emerge from the scutellar node at germination contemporaneously or soon after the primary root (Sass, 1977; Erdelska and Videvoncova, 1993; Hochholdinger, 2009). It is not completely clear whether primordia for all seminal roots are differentiated before germination (Feldman, 1994). Depending on genotypes and/or growing conditions, primary and seminal roots may persist throughout the life cycle of the plants or die out after the formation of the shoot-borne system (Hochholdinger, 2009). Intriguingly, seminal roots appear to be a unique feature of maize among Poaceae and, as such, do not develop in sorghum (Singh *et al.*, 2010).

Developmental genetics of seminal roots is incompletely known and seems to involve gene functions partially shared with other root organs. Mutants of the maize genes *rtcs* and *rum1*, which code for a LOB-domain and an Aux/IAA protein, respectively, abolish the initiation of seminal roots (Hetz *et al.*, 1996; Woll *et al.*, 2005; Taramino *et al.*, 2007; von Behrens *et al.*, 2011). Additionally, *rum1* also affects the initiation of lateral roots in the primary root, while *rtcs* abolishes the initiation of crown and brace roots. Recently, single nucleotide polymorphism (SNP) and/or haplotype variation at the same two genes across germplasm collections has been associated with phenotypic variation of seedling root traits (Kumar *et al.*, 2014; Zhang *et al.*, 2014).

In maize, variation in the seminal root number (SRN) is typically quantitative with a relatively strong genetic basis. The number of seminal roots ranges between 0 and 13 among maize accessions, with the lowest numbers more common in Flint-related genotypes (Kiesselbach, 1949; Burton *et al.*, 2013; Lynch, 2013). However, the range of variation was notably lower when elite materials were considered, ranging between 0 and ~7 seminal roots in a collection of 74 elite inbred lines (Kumar *et al.*, 2012) or between two and four in a collection of 19 elite F_1_ hybrids (Ali *et al.*, 2015). Fewer seminal roots were detected in teosinte as compared with maize (Burton *et al.*, 2013). Quantitative trait locus (QTL) mapping studies exploiting experimental cross-populations mostly showed a relatively low number of SRN QTLs (Tuberosa *et al.*, 2002b; Hund *et al.*, 2004; Zhu *et al.*, 2006; Trachsel *et al.*, 2009; Burton *et al*., 2014), suggesting either simple genetic control of the trait or reduced genetic variation in the investigated genetic backgrounds.

Seminal roots are important for the prompt establishment and early development of the seedling in the first 2 weeks from germination (Hochholdinger, 2009; Hochholdinger and Tuberosa, 2009). Accordingly, seminal roots are considered when maize ideotypes are designed (Lynch *et al.*, 2013). However, the actual mechanisms and extent by which the number and architecture of seminal roots would impact maize crop performance in terms of final yield is still a matter of debate.

In this work, we studied the genetic basis of variation for maize embryonic root system architecture in a collection of introgression lines developed from the cross between B73, an important elite line, and Gaspé Flint, an extremely early landrace (Salvi *et al.*, 2011). We addressed the striking difference in number of seminal root between B73 and Gaspé Flint, which typically develop three and virtually no seminal roots, respectively. The information and plant materials developed in this work will support a more informed modeling of root ideotypes while allowing for a more effective deployment of the root QTLome (Salvi and Tuberosa, 2015) in order to enhance yield and/or yield stability in maize grown in environments with low nutrient and water availability.

## Materials and methods

Seed of the B73×Gaspé Flint introgression library (IL) (Salvi *et al.*, 2011) and of the two parental lines were utilized as source material. B73 is an elite inbred line belonging to the Iowa Stiff Stalk Synthetic heterotic group and is the maize genomic reference (Gerdes *et al.*, 1993; Schnable *et al.*, 2009). Gaspé Flint is a Canadian landrace belonging to the Northern Flint germplasm race group (Vigouroux *et al.*, 2008) and characterized by extreme earliness, low stature, high tillering, and multiple ears. The IL includes 75 lines obtained starting from the cross B73×Gaspé Flint and followed by five backcrosses using B73 as recurrent parent, and two cycles of selfing. Each IL line was shown to carry one or more Gaspé Flint homozygous introgression (i.e. substitutions; summing up to an average of 43 cM per line). Approximately 70% of the Gaspé Flint genome is represented in the IL collection (Salvi *et al.*, 2011). The simple sequence repeat (SSR)-based genotype matrix produced in Salvi *et al.* (2011) was utilized in this study.

The investigation of the architecture of the embryonic root system was based on two independent experiments. In the first experiment, we utilized the paper roll (also known as paper cigar) technique as previously described (Hetz *et al.*, 1996; Woll *et al.*, 2005), with minor modifications as follows. Seeds were surface sterilized by immersion in 5% sodium hypochlorite solution for 10min followed by abundant rinsing with tap water, and pre-germinated for 2 d at 28 °C in the dark. Seedlings were transferred to rolls of filter paper (25×50cm dimensions), nine seedlings per roll. One roll with nine plants represented the experimental unit. Rolls were placed in 3 liter beakers with 400ml of deionized water and kept vertically for 7 d at 25 °C in the dark. Two replications (equivalent to two rolls per IL line) were carried out. At the end of the growing period, rolls were opened and visually scored for seminal root number (SRNppr), total length of seminal roots (SRLTppr), primary root length (PRLppr), and shoot length (STLppr). Additionally, digital images were recorded after moving seedlings from each roll to an A3-sized flat-bed scanner. Subsequently, the dry weight of seminal roots (SRDWTppr), primary roots (PRDWppr), and shoots (STDWppr) was obtained. In addition, the following traits were computed: embryonic root dry weight (ERDWppr)=(SRDWppr+PRDWppr); average seminal root dry weight (SRDWAppr)=(SRDWppr/SRNppr); and average seminal root length (SRLAppr)=(SRLTppr/SRNppr). In the second experiment, plants were grown in pots (5 liter volume, 50% sand, 50% clay pebbles) that were placed in a greenhouse. Pots were sown with three seedlings and then thinned to one seedling per pot at emergence. Pots were arranged in rows of five pots per IL line (five pots=one experimental unit) and two replications were performed. Pots were irrigated every second day and fertilized every 4 d with a half-strength Hoagland solution (Hoagland and Arnon, 1950). Plants were grown for 25 d, then uprooted, thoroughly washed, and subjected to manual/visual phenotyping. Traits collected or computed were SRNpt, STLpt, and STDWpt as described for the paper roll experiment. Samples for ERDWpt were obtained by cutting the seedling stems at the mesocotyl, just above the scutellum, in order to include both seminal and primary roots, which were not separated. In addition, both crown root number (CRNpt) and crown root dry weight (CRDWpt) were collected. The total number and dry weight of roots in pots (RNTpt and RDWTpt) were computed as follows: RNTpt=(1+SRNpt+CRNpt); RDWTpt=(ERDWpt+CRDWpt). Investigated traits are summarized in Table 1.

**Table 1.** Summary of the traits analyzed in this study, trait acronyms, and overview of phenotypic and genotypic variation within the B73×Gaspé Flint introgression library (IL)

Experimental system	Acronym	Trait	Unit	B73	Gaspé	IL (mean)	IL (min)	IL (max)	CV^*a*^	*h* ^*2*^ (%)	*P*-value lines (ANOVA)	*P*-value B73 versus Gaspé (Dunnett)	*P*-value normality (Shapiro)
Paper roll	ERDWppr	Embryonic root dry weight	mg	30.2	16.8	26.1	9.4	40.1	10.2	81	**	**	**
	PRDWppr	Primary root dry weight	mg	14.1	16.7	13.4	9.1	21.0	7.8	90	**	NS	**
	PRLppr	Primary root length	cm	30.6	37.7	30.0	25.7	35.3	5.1	70	**	**	NS
	SRDWAppr	Seminal root dry weight (average)	mg	5.7	0.0	4.9	1.4	7.0	8.0	93	**	**	**
	SRDWTppr	Seminal root dry weight (total)	mg	16.1	0.0	12.7	0.3	19.1	11.6	94	**	**	**
	SRLAppr	Seminal root length (average)	cm	25.3	0.0	22.7	8.8	29.3	11.1	81	**	**	**
	SRLTppr	Seminal root length (total)	cm	71.0	0.0	58.3	2.1	87.8	10.0	95	**	**	**
	SRNppr	Seminal root number	no.	2.9	0.0	2.5	0.2	3.7	8.7	95	**	**	**
	STDWppr	Shoot dry weight	mg	33.0	23.8	30.1	20.2	38.4	7.0	86	**	**	NS
	STLppr	Shoot length	cm	15.2	9.4	14.2	10.5	17.3	6.8	72	**	**	NS
Pot	CRDWpt	Crown root dry weight	mg	174.0	96.0	132.0	72.0	191.0	10.9	83	**	**	NS
	CRNpt	Crown root number	no.	7.5	7.2	6.8	5.5	8.1	6.4	72	**	NS	NS
	ERDWpt	Embryonic root dry weight	mg	141.0	100.0	122.0	64.0	170.0	11.4	78	**	NS	NS
	RDWTpt	Root dry weight (total)	mg	315.3	196.0	254.0	136.0	361.0	13.1	71	**	NS	NS
	RNTpt	Root number (total)	no.	10.3	7.4	8.9	7.0	11.1	14.2	69	**	**	NS
	SRNpt	Seminal root number	no.	2.9	0.1	2.3	0.1	3.0	12.0	88	**	**	**
	STDWpt	Shoot dry weight	mg	276.8	255.4	221.5	139.0	326.8	9.7	84	**	NS	NS
	STLpt	Shoot length	cm	28.2	21.5	26.9	22.6	32.2	4.8	75	**	**	NS

^*a*^ Coefficient of variation.

**P< 0.01; NS, not significant

Normality of frequency distribution was tested using the Shapiro–Wilk test and treated for normalization using the Box–Cox transformation (λ parameter set to default values) as implemented in Past 3.0 (Hammer *et al.*, 2001). ANOVA and correlation analysis among traits were carried out using R version 3.0.2 (R Core Team, 2013). Contrasts between B73, Gaspé Flint, and other IL lines trait values were carried out by Dunnett’s test. Heritability (*h* ^2^) was estimated following Falconer and Mackay (1996) using:

$$h^{2}=\frac{\sigma_{\text{G}}^{2}}{\sigma_{\text{G}}^{2}+\frac{\sigma_{\text{E}}^{2}}{r}}$$

where σ^2^_G_ and σ^2^_E_ represent the genotypic and the environmental components of the phenotypic variance, respectively, and *r* the number of replications (with *r*=2 for each of the two experiments, paper roll and pot).

QTL analysis was carried out with QTL IciMapping v4.0 (Meng *et al.*, 2015) using the method ‘Likelihood ratio test based on stepwise regression for additive QTL’ (RSTEP-LRT-ADD) which specifically deals with QTL mapping in collections with multiple introgressions per line. The algorithm implementing RSTEP-LRT-ADD was initially described in Wang *et al.* (2006). Parameters setting was ‘Multicollinearity control’= −1 (equivalent to deletion of duplicated markers only) and ‘PIN’=0.0001 (PIN: the largest *P*-value for entering variables in stepwise regression of residual phenotype on marker variables). Logarithm of odds (LOD) threshold values for each trait were computed after 10 000 permutations using a type I error=0.05 and are reported in Table 3 footnotes. The proportion of variance explained by a single QTL was as provided from simple marker analysis in RSTEP-LRT-ADD. The proportion of variance explained by all QTLs for number of seminal roots in the paper roll was computed by a multiple regression model using the markers *umc1685*, *umc1528*, and *Vgt1*-*mite* as independent variables.

## Results

### Phenotypic observations, variance component analysis, and trait distributions

Variation for seminal roots traits was analyzed using two different experimental systems, namely paper rolls and sand-filled pots. A total of 18 traits (10 from the paper roll and eight from the pot experiment, respectively) were collected, including 14 root- and four shoot-related traits. Variation for all traits was shown to be under clear genetic control (*P* ANOVA <0.01. Table 1), with *h* ^2^ values ranging from 0.69 to 0.95 (Table 1).

**Fig. 1.**
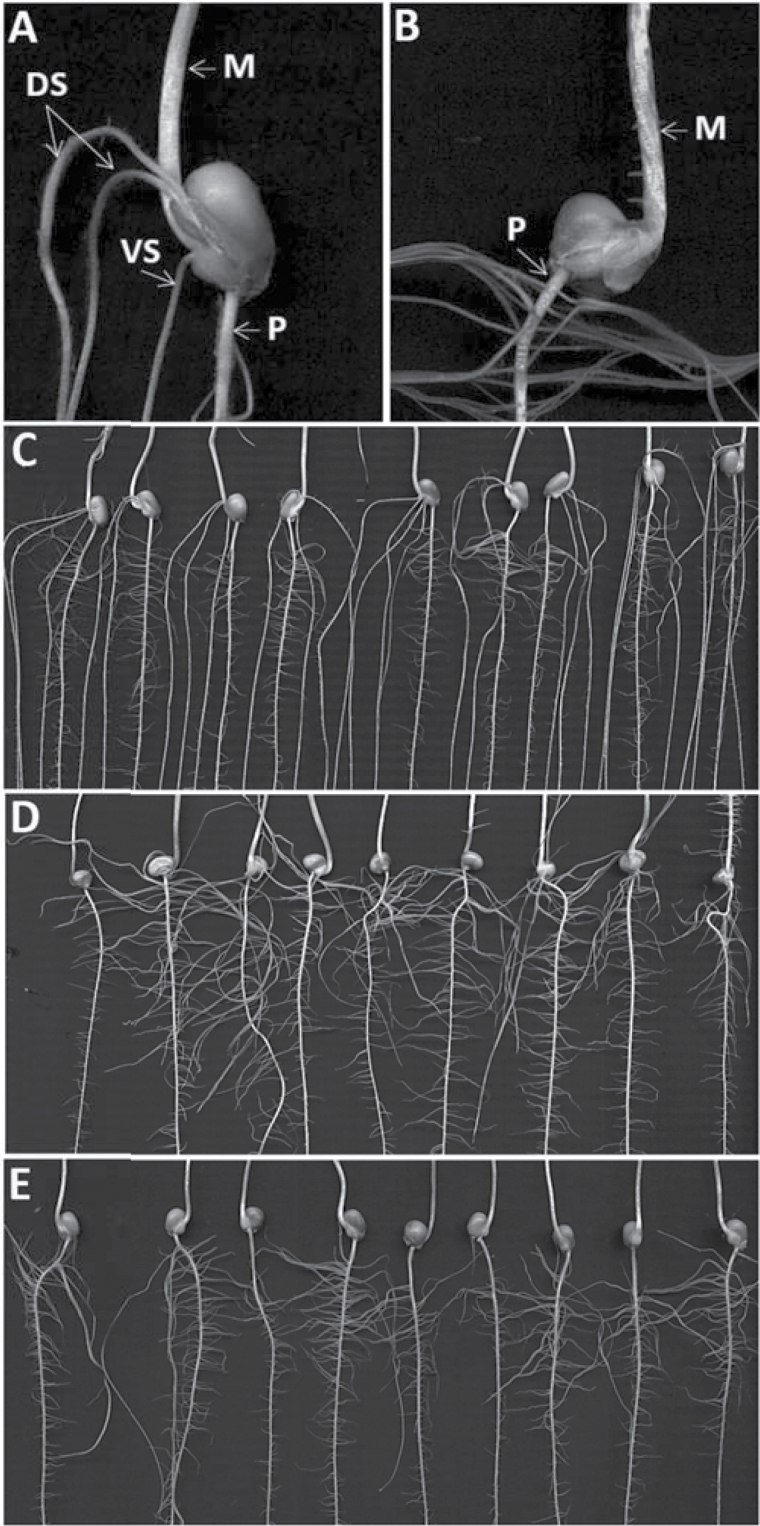
(A and B) Seminal root architecture of parental lines B73 and Gaspé Flint, respectively. DS, dorsal seminal roots; VS, ventral seminal root; M, mesocotyl; P, primary root. (C–E) Images of B73, Gaspé Flint, and line IL 94-6-1-6 root system architecture, respectively, as collected using the paper roll method

The two parental lines, B73 and Gaspé, showed striking differences for most root and shoot traits (Fig. 1A–D). Across experimental systems, B73 developed approximately three seminal roots (SRN=2.88±0.13) while seminal roots were almost completely absent in Gaspé (SRN=0.03±0.08). Conversely, Gaspé showed a significantly longer primary root than B73 in the paper roll system (PRLppr, Table 1). B73 showed a significantly higher embryonic root weight (seminal+primary) than Gaspé in paper rolls, but not in pots. In pots, B73 and Gaspé did not differ in the number of crown roots (CRNpt, 7.5 versus 7.2) although B73 crown roots were much heavier (CRDWpt, 174 versus 96mg) than those of Gaspé. As a consequence, and according to the paper roll-based results, B73 showed significantly higher dry weight of the whole root system than Gaspé (RDWTpt, 315 versus 96mg; *P*<0.01, Dunnet’s test).

Roots developing from the mesocotyl (Fig. 1B) were observed in 41% of Gaspé seedlings (total of 69 seedlings), while they were never observed in B73. Only one IL line (IL 55-14-1-3-7) occasionally showed seedlings with rooted mesocotyls (15% of seedlings; not shown).

B73 showed significantly longer and heavier shoots than Gaspé both in pots and in paper rolls, with the exception of shoot dry weight in pots (Table 1).

The frequency distribution and relative performance of IL lines versus parent lines differed among traits (Table 1; Supplementary Fig. S1 at *JXB* online). Traits related to the number, length, and dry weight of seminal roots (SRNppr and SSRpt, SRDWAppr, SRDWTppr, SRLAppr, etc.) showed slightly non-normal distributions skewed toward the B73 values. Specifically, SRN ranged between 0.2 and 3.7 and between 0.1 and 3.0 in the paper roll and pot experiment, respectively. One example of a B73 near isogenic IL line (IL 94-6-1-6) showing an extreme Gaspé-like phenotype (reduced number of seminal roots) is provided in Fig. 1E. No transgression of IL lines beyond parental values was observed for these traits. A similar distribution was observed for primary root-related traits (PRDWppr and PRLppr), in this case with B73 showing lower values than Gaspé. No IL line showed primary root length (PRLppr) longer than Gaspé. Crown roots and shoot-related traits showed frequency distributions not significantly different from normal (Table 1).

### Seminal root architecture

Detailed observations based on the paper roll system showed a highly consistent seminal root architecture in B73, with two seminal roots developing from between the mesocotyl and the seed (or dorsal seminal roots; Erdelska and Vidovencova, 1993) and one additional seminal root protruding out frontally from the scutellar node (or ventral seminal root; Fig. 1A). Given the observed strong segregation for SRN within the IL collection (from 0.2 to 3.7 seminal roots in the paper roll experiment), we wondered whether this range of SRN was associated with specific root architectural types. For instance, seedlings with three seminal roots could carry two dorsal seminal roots and one ventral root (summarized as type D2V1), like the typical B73 architecture, or could carry three dorsal seminal roots and no ventral root (type D3V0), or seedlings could even be of type D1V2 or D0V3. Table 2 summarizes the results linking the number of seminal roots to the architectural types as observed in two replicates in the paper roll experiment. Among seedlings with one seminal root, type D1V0 was much more frequent than the opposite D0V1 (83% versus 17%). Among seedlings with two seminal roots, the most frequent type was D2V0 followed by D1V1 and D0V2 (75, 24, and 1%, respectively). Among seedlings with three seminal roots, the B73 type (i.e. D2V1) was by far the most prevalent (98%). Among seedlings with four seminal roots, the only two types observed were D3V1 and D2V2, with the latter prevalent among the IL lines (27% versus 73%), and a similar frequency among B73 seedlings (50% versus 50%). Altogether these results indicated that seminal roots do not develop dorsally or ventrally of the scutellum at random; instead they seem to develop following a specific pattern. The seedlings appear to prioritize the development of up to two dorsal seminal roots, while the third root is usually the ventral one. The position of the fourth seminal root does not appear to be as tightly fixed, although type D2V2 was prevalent among IL lines. This proposed developmental pattern was confirmed when the SRN QTL genotype in single IL lines was considered (see QTL results). For example, IL lines with approximately two seminal roots (e.g. lines carrying the Gaspé allele at *qSRN-3.7*) almost invariably showed type B2F0; IL lines with approximately one seminal root (e.g. IL lines carrying *qSRN-1.2* or *qSRN-8.5* as homozygous for the Gaspé allele) were mostly of the D1V0 type. In other words, the three different SRN QTLs acted similarly within the constraints of the above-specified developmental pattern.

**Table 2.** Frequency of different seminal root architectures The prevalent architectural types are in bold. Data are from two replicates using the paper roll system

Number of seminal roots			IL (seedlings)		B73 (seedlings)	
Total	Dorsal	Ventral	*n*	%	*n*	%
4	4	0	0	0.0	0	0.0
	3	1	15	26.8	3	50.0
	**2**	**2**	**41**	**73.2**	**3**	**50.0**
	1	3	0	0.0	0	0.0
	0	4	0	0.0	0	0.0
3	3	0	5	0.9	0	0.0
	**2**	**1**	**522**	**97.9**	**90**	**100.0**
	1	2	6	1.1	0	0.0
	0	3	0	0.0	0	0.0
2	**2**	**0**	**270**	**75.4**	**16**	**76.2**
	1	1	85	23.7	5	23.8
	0	2	3	0.8	0	0.0
1	**1**	**0**	**43**	**82.7**	**0**	**0.0**
	0	1	9	17.3	0	0.0

### Correlation between traits

As expected, positive correlations were observed between traits related to the presence of seminal roots such as number, length, and dry weight of seminal roots from both paper roll and pot systems (*r* ranging from 0.43 to 0.95, *P*<0.01; Supplementary Table S1). Interestingly, the numbers of seminal roots from both the paper roll and pot plants were negatively correlated with primary root dry weight (*r*= −0.52 and −0.50, respectively; *P*<0.01), and similar albeit milder correlations were recorded with primary root length. Seminal root number was not correlated with brace root and shoot traits. Brace root number and dry weight, which were collected only in the pot experiment, positively correlated with shoot length and dry weight in the pot (*r*= 0.44–0.83; *P*<0.01) but showed only limited positive correlation with shoot length and dry weight in the paper roll (*r*=0.18–0.35; *P* from non-significant to <0.01).

Additionally, we tested the correlation between root traits and flowering time using flowering time data for the same IL lines from a previous study (Salvi *et al.*, 2011). Notably, traits related to seminal root number (ERDWppr, SRDWTppr, SRLAppr, SRLTppr, SRNppr, and SRNpt) positively correlated with flowering time [we report the correlation value for node number (ND), a highly heritable proxy for flowering time in maize; Salvi *et al.* (2011)]. Correlation between seminal root traits and ND ranged from 0.35 to 0.53 (all *P*-values <0.01). Shoot length in the paper roll system was also positively associated with ND (*r*=0.25; *P*<0.05).

Finally, a weak correlation was observed between root and shoot traits analyzed in this study and seed weight as collected on the original batch of seeds used for the paper roll and pot experiments (Supplementary Table S1). The only marginally statistically significant correlations with seed weight were observed with embryonic root dry weight in the paper roll and pot systems (ERDWppr and ERDWpt), and shoot dry weight in the pot system (STDWpt), with *r*=0.20, 0.18, and 0.23, respectively (*P*=0.05, 0.09 and 0.03, respectively).

### QTL results

**Fig. 2.**
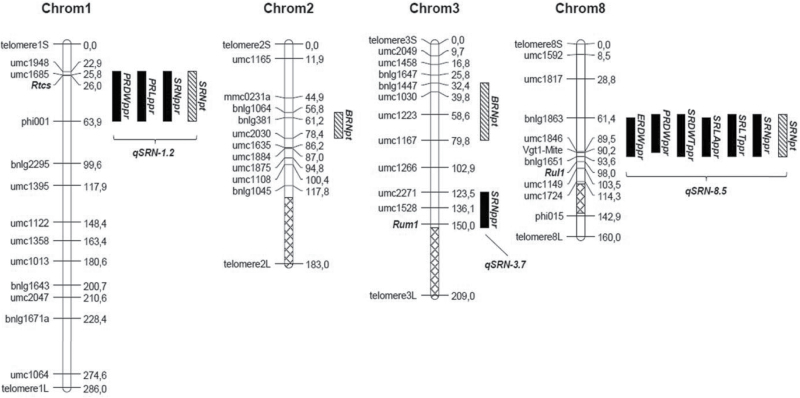
Map position of QTLs for root traits as identified in both the paper roll and pot experiments in this study. Chromosome maps were drawn based on SSR markers utilized in this study, and markers were positioned accordingly with the maize ‘Genetic’ maps available at MaizeGDB (www.maizegdb.org/data_center/map) using MapChart. The position of three genes or mutants thought or known to be involved in seminal root development (*rtcs* on chr. 1, *rum1* on chr. 3, and *rul1* on chr. 8) were added to the graph based on their position on the maize ‘Genetic’ map. Black and ticked bars represent the confidence interval for QTLs from the paper roll and pot experiments, respectively. Cross-ticked portion segments on chromosomes bars represent non-informative regions (because of lack of introgression in the collection or absence of marker polymorphisms (see Salvi *et al.*, 2011 for details).

Given the high coincidence of QTLs from the paper roll and pot experiments, and the relatively simple genetic control of the traits, overlapping QTLs for different traits will be presented and analyzed jointly. Three chromosome regions, corresponding to bins 1.02, 3.07, and 8.03–8.05, were shown to carry QTLs for seminal root traits (Table 3; Fig. 2). A QTL controlling the number of seminal roots, *qSRN-1.2*, was identified in both the paper roll and pot systems, and peaked at marker *umc1685* (25.8 cM position, on chr. 1). Considering both the paper rolls and pots, the genetic effect (*a*) at this QTL ranged from –0.66 to –0.83 roots (with the Gaspé allele decreasing the trait value), corresponding to ~24–30% of B73 seminal root number. A QTL of similar effect in terms of both intensity and direction of genetic effect, *qSRN-8.5*, was identified on chr. 8 in the interval between 58.7 cM and 93.6 cM encompassing a portion of bin 8.03 and bins 8.04 and 8.05. Markers near the QTL peaks were *Vgt1* (90.2 cM) or *Bnlg1863* (61.4 cM), in the paper roll and pot experiments, respectively. At this chromosome region, QTLs for other traits were mapped, such as SRDWTppr, SRDLTppr, SRLAppr, and ERDWppr. The direction and intensity of genetic effects at these QTLs supported the notion that the effect of *qSRN-8.5* on the number of seminal roots was the main driver of the observed variation (see Discussion). In the paper roll experiment only, one additional QTL for SRN, *qSRN-3.7*, was mapped at bin 3.07, with *umc1528* (136.1 cM) as the peak marker and with genetic effect *a*= −0.61 roots. Although the *qSRN-3.7* LOD score was below the threshold (LOD *qSRN-3.7*=3.2 versus LOD_*P*<0.05_ threshold=3.6), the concordance of the computed QTL position with the observed mean values of IL lines carrying Gaspé chromosome introgressions in the same region (not shown) and the coincidence with SRN QTLs in other studies (see Discussion and Supplementary Table S2) suggest that this QTL is real. The three SRN QTLs explained 66% of phenotypic variance when considered in a multiple regression model.

**Table 3.** Summary of all detected QTLs for embryonic and seedling traits analyzed in this study

QTL	Trait^*a*^	Marker^*b*^	Bin	Position^*c*^	LOD^*d*^	PVE^*e*^ (%)	Effect^*f*^ (a)	Effect^*g*^ (% B73)
*qSRN-1.2*	SRNppr	*umc1685*	1.02	22.9 - 25.8 - 63.9	3.63	20.7	–0.83 roots	–29.5
	SRNpt	*umc1685*	1.02	22.9 - 25.8 - 63.9	3.97	22.7	–0.66 roots	–23.6
	*PRDWppr*	*umc1685*	1.02	22.9 - 25.8 - 63.9	2.70	15.3	2.71 mg	19.3
	PRLppr	*umc1685*	1.02	22.9 - 25.8 - 63.9	2.70	16.1	2.40 cm	7.9
*qSRN-3.7*	*SRNppr*	*umc1528*	3.07	123.5 - 136.1 -152.8	3.20	18.4	–0.61 roots	–21.8
*qSRN-8.5*	SRNppr	*bnlg1863*, *Vgt1*, *umc1846*	8.05	58.7 - 90.2 - 93.6	5.47	29.5	–0.86 roots	–30.7
	SRNpt	*bnlg1863*, *Vgt1*, *umc1846*	8.03	58.7 - 61.4 - 93.6	4.04	23.0	–0.52 roots	–18.7
	ERDWppr	*Vgt1*, *umc1846*	8.05	61.4 - 90.2 - 93.6	4.05	23.1	–4.60 mg	–15.3
	PRDWppr	*bnlg1863*	8.03	58.7 - 61.4 - 90.2	3.83	21.9	1.93 mg	13.7
	SRDWTppr	*bnlg1863*, *Vgt1*, *umc1846*	8.05	58.7 - 90.2 - 93.6	6.39	33.9	–5.57 mg	–34.6
	SRLAppr	*Vgt1*, *umc1846*	8.05	61.4 - 90.2 - 93.6	4.92	27.3	-4.77 cm	-18.9
	SRLTppr	*bnlg1863*, *Vgt1*, *umc1846*	8.05	58.7 - 90.2 - 93.6	7.46	38.3	-25.33 cm	-35.7
*qCRN-2.4*	CRNpt	*bnlg381*	2.04	56.8 - 61.2 - 78.4	2.72	16.4	0.49 roots	6.5
*qCRN-3.4*	CRNpt	*umc1030*, umc1223	3.04	32.4 - 58.6 - 79.8	3.36	19.8	0.62 roots	8.2

^*a*^ -ppr, paper roll experiment; -pt, pot experiment.

^b^ For each QTL, all adjacent markers significantly associated with phenotypic variation, and concordant in direction of genetic effect, are reported. In the case of multiple adjacent markers, the marker with the highest LOD value is underlined.

^*c*^ From left to right, the three cM values indicate the positions of: the QTL nearest upper marker which was not statistically significant, the marker with the highest LOD score; and the QTL nearest lower marker which was not statistically significant.

^*d*^ LOD thresholds (*P*<0.05 based on permutation) were: SRNppr, 3.5; SRNpt, 3.9; PRDWppr, 3.0; PRLppr, 2.7; ERDWppr, 2.5; SRDWTppr, 3.5; SRLAppr, 3.6; SRLTppr, 3.7; CRNpt, 2.4; CRDWpt, 2.4.

^*e*^ PVE, proportion of phenotypic variance explained.

^*f*^ QTL genetic effect expressed as *a*=(mean GG–mean BB)/2, where GG represents lines homozygous for the Gaspé Flint allele and BB represent lines homozygous for the B73 allele.

^*g*^ QTL genetic effect expressed as percentage of B73 (parental line) trait value.

Based on the paper roll experiment, *qSRN-1.2* seemed to have an effect on primary root length (PRLppr), with the Gaspé Flint allele increasing the trait value (*a*=2.40cm, corresponding to 7.9% of the B73 trait value). This effect on PRLppr is in the opposite direction to that shown by the same QTL on SRNppr where the Gaspé allele decreased the trait value. A similar, albeit non-significant, effect (*P*<0.1) was observed on primary root dry weight (PRDWppr). The Gaspé Flint allele at *qSRN-8.5* which was characterized by a negative effect of SRN also showed a positive effect on PRDWppr (*a*=1.93mg, corresponding to 13.7% of the B73 trait value) but no significant effect was recorded on PRLppr. These observations suggest a physiological trade-off between seed resources to be devoted to primary or seminal roots.

Two QTLs for the number of crown roots in the pot experiment, *qCRN-2.4* and *qCRN-3.4*, were mapped on chrs 2 and 3 near peak markers *bnlg381* (61.2 cM) and *umc1223* (58.6 cM), respectively (Fig. 2). For both QTLs, the Gaspé allele showed a positive genetic effect of *a*=0.49 or 0.62 roots, respectively (Table 3). No QTLs were identified for the traits not mentioned here.

## Discussion

### Genetic control of seminal root architecture in B73×Gaspé Flint

The genetic control of the number of seminal roots in the cross B73×Gaspé Flint appeared rather simple, with just three major QTLs accounting for 66% of phenotypic variation and with Gaspé Flint contributing the trait-decreasing allele at all QTLs. Interestingly, two (*qSRN-1.2* and *qSRN-3.7*) of the three QTLs for number of seminal roots co-mapped with the only two maize genes known to affect the presence of seminal roots in maize, *rtcs* and *rum1*. *qSRN-1.2* (mapped at 22.9–63.9 cM) includes *rtcs* (at 26.0 cM, based on the MaizeGDB) within its confidence interval. The *rtcs* gene regulates seminal root initiation in the scutellar node and shoot-borne crown and brace roots in the shoot nodes; however it does not affect primary and lateral roots. As a consequence, *rtcs* mutants lack seminal and crown/brace roots (Hetz *et al.*, 1996). Notably, *rtcs* codes for a LOB-domain transcription factor and carries auxin-responsive elements in its promoter (Taramino *et al.*, 2007). At the seedling level, *qSRN-1.2* and *rtcs* show very similar effects. In particular, the *qSRN-1.2*-Gaspé allele showed a remarkable additive effect equal to −0.83 seminal roots, leading to less than one seminal root in lines homozygous for the Gaspé allele (versus almost three seminal roots observed in B73). Conversely, the phenotypic expression of *qSRN-1.2* and *rtcs* differed markedly at the adult stage. While IL lines carrying the *qSRN-1.2*-Gaspé allele develop standard adult crown/brace root apparatus (not shown), *rtcs* mutant plants are completely devoid of any shoot-borne root and require protection from dehydration and external support against lodging in order to reach maturity (Hetz *et al.*, 1996). Additionally, our data suggest that *qSRN-1.2* influences both the number and average length or weight of seminal roots. In other words, not only does differentiation seem to be impaired but also root elongation, which theoretically should entail rather different gene functions. However, recently a similar concerted control of both differentiation and elongation was demonstrated by *rtcs* and its closely related paralog *rtcl* (Xu *et al.*, 2015). Natural allelic variation among maize lines for *rtcs* was already demonstrated and associated with root phenotypic variation (Zhang *et al.*, 2013). Based on these observations, it is likely that *qSRN-1.2* and *rtcs* represent the same locus. As compared with B73, Gaspé Flint would carry a mildly diverged *rtcs* allele characterized by reduced gene or protein functionality or an altered pattern of gene expression specifically affecting seminal root development. The formal positional cloning of *qSRN-1.2* is in progress in our group.

*qSRN-3.7* (123.5–152.8 cM) overlaps with *rum1* (at 150 cM). The mutant *rum1* is impaired in the initiation of seminal roots and of lateral roots in the primary root (Woll *et al.*, 2005). *qSRN-3.07/8* simply showed a quantitative effect (slightly lower than *qSRN-1.2*) on a number of seminal roots and it did not affect lateral roots in the primary root (not shown). Similar to the *qSRN-1.2*–*rtcs* pair, *qSRN-3.7* could well be a mild mutant allele (in the regulation of either expression or coding/function) of *rum1*. *rum1* was found to be associated with seedling root trait variation in diverse collections (Kumar *et al.*, 2014; Pace *et al.*, 2015), in one case evaluated at different N levels (Abdel-Ghani *et al.*, 2015).

To the best of our knowledge, no obvious seminal root candidate gene maps within *qSRN-8.5*. However, the gene *rum1-like* (*rul1*), a paralog of *rum1* (Kumar *et al.*, 2014), maps at bin 8.06 at 98 cM, only a few centiMorgans away from this QTL. SNP variation within *rul1* was found to be associated with root dry weight in 10- and 14-day-old seedlings (Kumar *et al.*, 2014). Given the proximity of the QTL and this gene, further tests are required in order definitely to exclude *rul1* from being a candidate for this QTL.

The *qSRN-1.2/rtcs* region is relatively enriched with QTLs for root morphology and architecture, as identified in several experiments (Supplementary Table S2). However, the poor root type definition utilized in several studies, and/or the rather different experimental conditions (e.g. gel-based or field), prevented a more comprehensive comparison among QTL studies. Seventeen QTLs for different root architecture traits were previously mapped at this region in seven different experimental crosses involving 13 inbred lines (our analysis updating Hund *et al.*, 2011). However, only in four of such crosses and QTL analyses [Lo964×Lo106 in Tuberosa *et al.* (2002b) and Hund *et al.* (2004); B73×Mo17 in Zhu *et al.* (2006); CML444×SC-Malawi in Trachsel *et al.* (2009)] were seminal roots recognized and analyzed separately, while seminal roots were mixed with other root types in the remaining five crosses (Supplementary Table S2). Interestingly, the direction of QTL effects was consistent in different crosses or experiments. For example, the B73 allele consistently increased the number of seminal roots (Zhu *et al.*, 2006; this study). The increased root ‘bushiness’ detected for the B73 allele in Zurek *et al.* (2015) may well be due to a higher number of seminal roots and therefore also goes in this direction.

### A constrained seminal root developmental pattern

Seminal roots develop at the scutellar node mainly from behind and in front of the mesocotyl (Kiesselback, 1949; Singh *et al.*, 2010). Erdelska *et al.* (1990) distinguished between dorsal, ventral, and intermediate seminal roots based on the position of their primordia as observed in scutellar microscopy cross-sections. The dorsal primordia are the first ones to differentiate in the embryo within ~30 d after pollination, followed by ventral and then intermediate primordia (Erdelska and Vidovencova, 1993). The two dorsal primordia originate close to the main scutellar vascular system, on the scutellum endosperm-facing side (Hetz *et al.*, 1996), and give rise to the two seminal roots emerging from behind the mesocotyl. Our results confirmed that seminal roots do not emerge randomly at the scutellar node. The IL background line B73 almost constantly developed three seminal roots arranged in an invariable architecture characterized by two dorsal twin seminal roots developing in between the mesocotyl and the seed and one ventral seminal root protruding from the scutellar node and through the base of the mesocotyl. Among the collection of IL lines, irrespective of the final number of seminal roots (ranging from 0.1 to 3.7), the position of seminal roots followed a precise pattern, with priority given first to dorsal, then to ventral, and then to additional seminal roots, in accordance with the chronological sequence of appearance of seminal root primordia (Erdelska and Vidovencova, 1993). In other words, the dorsal seminal roots were only present when the total number of seminal roots was one or two, while the ventral seminal(s) appeared only in seedlings with more than two roots. The position of a fourth seminal root appeared less constrained as it could flank either the two dorsal roots or the ventral root. The three SRN QTLs did not act on the number of one specific type of seminal root only (i.e. just on the number of dorsal, or ventral, etc.) but rather on the total number of seminal roots.

Altogether, these observations implicate the existence of as yet unknown constraints that direct the differentiation of seminal roots to given scutellar districts. Our results also suggest that different SRN loci influence the total number of seminal roots without over-riding a developmental pattern that apparently gives priority to dorsal roots. It is plausible that the three SRN QTLs act on the level of auxin concentration (or, alternatively, auxin sensitivity) at competent scutellar cells following the observation that treatment with exogenous NAA (naphthalene acetic acid) induced the development of additional ‘adventitious’ seminal roots at/around the scutellar/mesocotyl region in *rtcs* mutants (Hetz *et al.*, 1996).

It would be interesting to verify the above-described pattern of seminal root architecture in diverse maize germplasm, also in view of the large diversity in seminal root number, and seed morphology and size. This information could be important to better tune new root ideotypes for breeding purposes, given the recently reported correlation between the growth angle of seminal roots, angle of nodal roots, and grain yield under drought conditions (Ali *et al.*, 2015).

### Clues on seminal root function and impact on crop performance

The seminal root system is commonly considered to be crucial for seedling survival and crop establishment (Sanguineti *et al.*, 1998; Hochholdinger, 2009; Tuberosa and Salvi, 2009; Lynch *et al.*, 2011). However, little experimental evidence is available on the effect of seminal root variation on maize crop performance. Unfortunately, the information which can be obtained by testing seminal root mutants such as *rtcs* or *rum1* does not enable the extrapolation of any effect of seminal roots on stress tolerance or yield performance due to the strong phenotypic expressivity/effect also involving crown, brace, or lateral roots (Hetz *et al.*, 1996; Woll *et al.*, 2005). In an early study, severing the seminal root system in 3-week-old maize plants caused a 9% reduction in yield (Kiesselback, 1949). A reduced P soil level was shown to increase average seminal root length and marginally to increase seminal root number (Zhu *et al.*, 2006), supporting the hypothesis of the importance of topsoil foraging in the early phase of maize development.

Correlation analysis between seminal root traits and complex agronomic traits expressed later in development, including yield, could theoretically provide information on the potential impact of seminal root variation. However, the complex quantitative variation for root system architecture along with the heterogeneous materials that were tested has so far prevented a meaningful evaluation of the effects of a difference in seminal roots. Not surprisingly, information from the literature often appears contradictory or not conclusive. Maize genotypes with vigorous root systems early in plant development were shown to have larger root systems at maturity (Nass and Zuber, 1971). Ali *et al.* (2015) showed that seminal root angle (collected at V1) correlated well with nodal root angle collected at a later stage (V6) in a collection of maize hybrids. However, the same authors showed that root angle correlated with grain yield in water stress conditions while the number of seminal roots did not. Some level of positive correlation between seedling root traits and shoot traits and even yield was detected in other studies (Richner *et al.*, 1997; Manavalan *et al.*, 2011); however, seminal roots were not distinguished from nodal roots. The number of seminal roots was negatively correlated with the root system volume in adult plants (Andrew and Solanki, 1966) and showed no or even negative correlation with field-based root traits such as root clumps or root-pulling force (Nass and Zuber, 1971). In Sanguineti *et al.* (2006), the weight of seminal roots was the only root seedling trait not negatively correlated with grain yield in a comparison of root and yield traits among a historical series of 47 maize hybrids released in the US Corn Belt from 1930 to 2000. Along this line, seminal root length was positively correlated with grain yield under both low and high N levels (Abdel-Ghani *et al.*, 2015), and seminal root QTLs consistently overlapped with grain yield QTLs (with concurrent direction of genetic effect) in one mapping population (Tuberosa *et al.*, 2002b) and in a meta-analysis (Tuberosa *et al.*, 2002a).

Our results clearly suggest a negligible correlation between SRN and both crown roots and shoot traits at the seedling stage. Results of QTL analyses supported this notion since crown root QTLs mapped independently from SRN QTLs, and no QTLs for shoot traits were identified. Thus, a mostly independent genetic control of seminal root traits relative to crown roots and shoot traits is likely. This would enable more precise manipulation of root ideotypes by acting on single root organs as recently proposed (Lynch, 2013).

As observed in other studies (Manavalan *et al.*, 2011; Abdel-Ghani *et al.*, 2015), we found little correlation between seed weight and root traits. The only two traits found to (mildly) correlate with kernel weight were root dry weight in paper roll and pot systems (ERDWppr and ERDWpt), and shoot dry weight in pot experiments (STDWpt), which are expected to be affected by the availability of seed resources in experiments with no (for paper roll) or minimal (for pots) external nutrient addition.

Linkage most probably significantly contributed to the observed positive correlation between SRN and flowering time. Two of the three SRN QTLs mapped at or near bins 3.07 and 8.05, where major flowering time QTLs have already been mapped, in this and other populations (Salvi *et al.*, 2009, 2011).

The current study also revealed additional interesting correlations within the embryonic systems. We observed a negative correlation between SRN and primary root dry weight (PRDWppr) or length (PRLppr). Results of the QTL analysis confirmed these observations since *qSRN-1.2* and *qSRN-8.5*, besides their strong effect on the number of seminal roots, affected primary root length and weight (respectively) with opposite direction of genetic effects [i.e. the allele that increased the number of seminal roots (namely, B73) also decreased primary root size]. In line with our results, in ‘t Zandt *et al.* (2015) observed a 2-fold increase in seminal root dry weight upon removal of primary root. The most likely explanation of these findings is the presence of a relocation mechanism for seed endosperm resources that apparently sustains root development irrespective of the genetic constraints on seminal roots. The stronger negative correlation of SRNppr with primary root dry weight rather than with primary root length may also suggest that the signal received by the primary root is more precisely interpreted in terms of developing or not developing lateral roots (which make up a sizeable fraction of root weight), unfortunately not collected in our study, and only marginally acting on primary root length. Alternatively, the effect on primary root weight could be due to a modulation of the level of primary root cavitation (e.g. level of aerenchyma; Lynch *et al.*, 2013) in relation to the availability of seed resource. Both mechanisms would eventually sustain the growth in length of the primary root, which is in line with its important role for adaptation and survival of the young seedling. Additionally, it should be noted that IL lines with a genetically determined low number of seminal roots (as compared with B73) redirected the seed resource to the primary root only, and not to the remnant seminal roots. This is clearly shown by the positive correlation between number of seminal roots and seminal root length or dry weight, either total or average (*r* ranging from 0.43 to 0.54. Supplementary Table S1). This rather unexpected result is probably due to the partially shared and/or interconnected action of genes such as *rtcs* and *rum1* on both differentiation and growth of seminal roots (Xu *et al.*, 2015), as already discussed.

### Conclusions

Variation in seminal root architecture in a dent×flint type of cross in maize appears to be under oligogenic control, with just three QTLs for number of seminal roots controlling most of the variation. Two out of three QTLs co-mapped with two genes that are among the few known to be involved in seminal root development. Based on the properties of the IL population utilized here for mapping, pairs of near isogenic lines differing for the target QTLs are immediately available (or can be prepared in a simple marker-assisted selection step) for downstream studies such as fine mapping and cloning the underlining QTLs and further functional investigations. More specifically, pairs of lines including B73 and its near isogenic version with reduced or null seminal roots were identified. This provides for the first time the opportunity to test unequivocally in an agronomically viable genetic background (provided by B73) how variation for seminal roots in maize affects seedling establishment and early plant growth, and eventually biomass and grain yield.

## Supplememtary data

Supplementary data are available at *JXB* online.

Figure S1. Frequency distribution for the 18 root and shoot seedling traits analyzed in the B73×Gaspé Flint introgression library (IL) population (75 IL lines) utilized in this study.

Table S1. Analysis of correlation among root traits collected in the two experiments (paper roll, -ppr; and pot, -pt).

Table S2. Root trait mutants and QTLs mapping near to (or overlapping with) the three QTLs for seminal root number (*qSRN-1.2*, *qSRN-3.7*, and *qSRN-8.5*) identified in this study

## Supplementary Material

Supplementary Data
