# Supplementary material for: Two major quantitative trait loci controlling the number of seminal roots in maize co-map with the root developmental genes rtcs and rum1
Source: J Exp Bot. 2016 Feb 13;67(4):1149–59. doi: 10.1093/jxb/erw011 (PMC4753855; doi:10.1093/jxb/erw011)
Supplement: Supplementary Data [file supp_67_4_1149__index.html]

Two major quantitative trait loci controlling the number of seminal roots in maize co-map with the root developmental genes rtcs and rum1 — Supplementary Data 

# Two major quantitative trait loci controlling the number of seminal roots in maize co-map with the root developmental genes *rtcs* and *rum1*

## Supplementary Data

Data files

- supplementary\_table\_S1\_S2\_figure\_S1.pdf - Supplementary Data
