# Supplementary material for: Two major quantitative trait loci controlling the number of seminal roots in maize co-map with the root developmental genes rtcs and rum1
Source: J Exp Bot. 2016 Feb 13;67(4):1149–59. doi: 10.1093/jxb/erw011 (PMC4753855; doi:10.1093/jxb/erw011)

Supplementary Table 1. *Analysis of correlation among root traits collected in the two experiments (paper-roll, -ppr; and pot, -pt). The table reports the r (Pearson) correlation value (below diagonal) and the corresponding P values (above diagonal)*

	BRDWpt	BRNpt	ERDWppr	ERDWpt	PRDWppr	PRLppr	RDWTpt	RNTpt	STLppr	STLpt	STDWppr	STDWpt	SRDWAppr	SRDWTppr	SRLAppr	SRLTppr	SRNppr	SRNpt	ND	SeedW
BRDWpt		0.00	0.03	0.00	0.00	0.00	0.00	0.00	0.03	0.00	0.00	0.00	0.11	0.45	0.18	0.76	0.53	0.91	0.29	0.09
BRNpt	0.64		0.17	0.00	0.35	0.44	0.00	0.00	0.04	0.00	0.14	0.00	0.28	0.34	0.53	0.55	0.44	0.10	0.42	0.15
ERDWppr	0.27	0.17		0.01	0.03	0.06	0.00	0.00	0.15	0.11	0.00	0.01	0.00	0.00	0.00	0.00	0.00	0.00	0.00	0.05
ERDWpt	0.72	0.43	0.32		0.22	0.01	0.00	0.00	0.06	0.00	0.00	0.00	0.00	0.03	0.01	0.08	0.18	0.04	0.03	0.09
PRDWppr	0.37	0.12	0.26	0.15		0.00	0.02	0.22	0.48	0.14	0.00	0.07	0.34	0.03	0.79	0.00	0.00	0.00	0.08	0.47
PRLppr	0.38	0.10	0.23	0.31	0.48		0.00	0.56	0.18	0.00	0.00	0.00	0.07	0.84	0.10	0.48	0.04	0.16	0.82	0.20
RDWTpt	0.93	0.59	0.34	0.91	0.29	0.38		0.00	0.04	0.00	0.00	0.00	0.01	0.09	0.02	0.21	0.63	0.23	0.06	0.56
RNTpt	0.42	0.77	0.35	0.44	-0.15	-0.07	0.49		0.14	0.00	0.57	0.00	0.00	0.00	0.00	0.00	0.00	0.00	0.04	0.20
STLppr	0.26	0.25	0.17	0.23	0.09	0.16	0.26	0.18		0.66	0.00	0.18	0.78	0.28	0.25	0.13	0.39	0.28	0.04	0.18
STLpt	0.68	0.43	0.20	0.64	0.18	0.35	0.72	0.34	0.05		0.10	0.00	0.09	0.36	0.39	0.84	0.73	0.71	0.88	0.21
STDWppr	0.35	0.18	0.55	0.36	0.52	0.37	0.36	0.07	0.56	0.20		0.01	0.00	0.02	0.02	0.12	0.59	0.56	0.51	0.12
STDWpt	0.83	0.49	0.32	0.88	0.22	0.39	0.93	0.42	0.17	0.73	0.34		0.01	0.07	0.01	0.18	0.69	0.21	0.14	0.03
SRDWAppr	0.20	0.13	0.86	0.35	0.11	0.21	0.31	0.36	0.03	0.21	0.37	0.33		0.00	0.00	0.00	0.00	0.00	0.01	0.24
SRDWTppr	0.09	0.12	0.87	0.26	-0.26	-0.02	0.21	0.45	0.13	0.11	0.28	0.22	0.80		0.00	0.00	0.00	0.00	0.00	0.22
SRLAppr	0.16	0.08	0.75	0.33	-0.03	0.20	0.28	0.36	0.14	0.11	0.27	0.30	0.90	0.76		0.00	0.00	0.00	0.00	0.68
SRLTppr	0.04	0.08	0.76	0.22	-0.38	-0.08	0.16	0.46	0.18	0.02	0.19	0.17	0.68	0.95	0.77		0.00	0.00	0.00	0.29
SRNppr	-0.08	0.10	0.59	0.16	-0.52	-0.24	0.06	0.49	0.10	-0.04	0.06	0.05	0.43	0.86	0.49	0.91		0.00	0.00	0.18
SRNpt	-0.01	0.20	0.44	0.24	-0.50	-0.17	0.15	0.72	0.13	0.05	-0.07	0.15	0.44	0.70	0.54	0.78	0.80		0.00	0.15
ND	0.13	0.10	0.43	0.27	-0.22	0.03	0.24	0.25	0.25	0.02	0.08	0.18	0.30	0.52	0.35	0.53	0.52	0.39		0.71

SeedW	0.21	0.14	0.23	0.18	-0.10	-0.17	0.04	0.12	0.18	0.15	0.19	0.23	0.14	0.16	0.10	0.12	0.16	0.15	-0.09	

Supplementary Table 2. *Root traits mutants and QTLs mapping near to (or overlapping with) the three QTLs for seminal root number (qSRN-1.2, qSRN-3.7, and qSRN-8.5) identified in this study*

QTL	Chr	CI [a] Left	CI [a] Right	Trait	Experimental system	Parents	Cross type	Reference
Osman_2013_rl1-2	1	16.12	49.49	Total root length	Pots	HZ32 x K12	F2:3	Osman et al. 2013
Salvi_qSRN-1.2	**1**	**22.90**	**63.90**	**Number of seminal roots**	**Paper roll**	**B73 x Gaspé**	**IL**	**This work**
Trachsel_2009_NoAx_1_3	1	23.00	53.00	Number of seminal roots	Pouches	CML444xSC-Malawi	RIL	Trachsel et al. 2009
Tuberosa_2002_R1L_1_34	1	25.30	70.88	Length of primary root	Hydroponics	Lo964 x Lo1016	F2:4	Tuberosa et al. 2002
Osman_2013_rl1-3	1	25.79	54.95	Total root length	Pot	HZ32 x K12	F2:3	Osman et al. 2013
rtcs	1	26.00	26.00	Number of seminal and crown/brace roots		Mutant		Taramino et al. 2007
Liu_2008_MaxLAx_1	1	36.29	52.06	Length of axial roots	Hydroponics	Z3 x 87-1	RIL	Liu et al 2008
Cai_2012a_LAx_1	1	38.05	69.54	Length of axial roots	Field	Ye478 x Wu312	BC4F3	Cai et al. 2012
Landi_2002_RPF_1	1	41.45	76.00	Vertical root pulling force	Field	Lo964 x Lo1016	F2:4	Landi et al. 2002
Tuberosa_2002_R1W_1_34	1	41.63	54.55	Weight of primary root	Hydroponics	Lo964 x Lo1016	F2:4	Tuberosa et al. 2002
Hund_2004_SeAx_Length_1_47	1	49.77	82.30	Length of seminal roots	Sand-vermiculite substrate	Lo964 x Lo1016	F2:4	Hund et al. 2004
Liu_2011_RPF_5	1	50.92	83.13	Vertical root pulling force	Field	Ye478 x Wu312		Liu et al. 2011
Hund_2004_SeAx_Count_1_49	1	51.30	87.15	Number of seminal roots	Sand-vermiculite substrate	Lo964 x Lo1016	F2:4	Hund et al. 2004
Hund_2004_PrAx_Diameter_1_44	1	53.05	72.06	Primary root diameter	Sand-vermiculite substrate	Lo964 x Lo1016	F2:4	Hund et al. 2004
Messmer_2006_RCT_9	1	55.00	76.00	Root capacitance	Field	CML444 x SC-Malawi		Messmer 2006
Burton_2014-SolPrinLen_Bin0228	1	60.00	70.00	Length of 2nd laterals, primary root	Pots with peat/vermiculite	OhxW64a	RIL	Burton et al. 2014
Tuberosa_2002_R2W_1_46	1	62.41	73.89	Weight of seminal roots	Hydroponics	Lo964 x Lo1016	RIL	Tuberosa et al. 2002
Zhu_2006_HighP_SRN1	1	5.70	28.60	Number of seminal roots	Paper roll	B73 x Mo17	RIL	Zhu et al. 2006
Ruta_2010_LRt_2	3	121.10	133.16	Length of lateral roots	Pouches	Ac7643 x Ac7729/TZSRW	RIL	Ruta et al. 2010
Salvi_qSRN-3.7	**3**	**123.50**	**152.80**	**Number of seminal roots**	**Paper-roll**	**B73 x Gaspé Flint**	**IL**	**This work**
Zhu_2006_NoSe_3	3	124.70	135.18	Number of seminal roots at high P	Paper-roll	B73 x Mo17	RIL	Zhu et al. 2006

Zhu_2005_LowP_LateralRootLength	3	128.66	131.22	Length of lateral roots at low P	Paper-roll	B73 x Mo17	RIL	Zhu et al. 2005
Liu_2011_VRPR_3	3	148.49	159.02	Vertical root pulling resistance	Field	Ye478 x Wu312	RIL	Liu et al. 2011
rum1	3	150.00	150.00	Number of seminal and primary lateral roots		Mutant		Woll et al. 2005
Hund_2004_PrAxDiameter_4	3	151.42	198.51	Diameter of primary root	Sand-vermiculite substrate	Lo964 x Lo1016	F2:4	Hund et al. 2004
Trachsel_2009_LPrAX_8	8	51.00	78.00	Length of primary root	Pouches	CML444 x SC-Malawi	RIL	Trachsel et al. 2009
Salvi_qSRN-8.5	**8**	**58.70**	**93.60**	**Number of seminal roots**	**Paper roll**	**B73 x Gaspé Flint**	**IL**	**This work**
Zhu_2005_LowP_LateralRootLength	8	60.88	62.77	Length of lateral roots at low P	Paper-roll	B73 x Mo17	RIL	Zhu et al. 2005
Barriere_2001_AnCr_1	8	62.20	70.44	Angle of crown roots	Field	F288 x F271	RIL	Barriere et al. 2001
Tuberosa_2002_R1L_8_48	8	70.75	90.24	Length of primary root	Hydroponics	Lo964 x Lo1016	F2:4	Tuberosa et al. 2002
Burton_2014_SemNum bin_6786	8	82.00	83.00	Number of seminal roots	Pots with peat/vermiculite	B73 x M017	IRIL	Burton et al. 2014
Zurek_2015_maximumnumberofroots_d06	8	87.10	98.00	Number of roots	Gel-based rhizotrons	B73 x Ki3	RIL	Zurek et al. 2015

[a] CI = QTL confidence interval, with left and right borders as obtained by projecting the original QTL confidence interval on the maize 'Genetic' map (available at the MaizeGDB, section Maps, http://www.maizegdb.org/data_center/map), using common markers as anchor points. *rtcs* and *rum1* cM positions are from the same map.

References cited in Supplementary Table 2

Barriere Y, Gibelin C, Argillier O et al (2001) Genetic analysis in recombinant inbred lines of early dent forage maize. I: QTL mapping for yield, earliness, starch and crude protein contents from per se value and top cross experiments. Maydica, 46, 253-266.

Burton AL, Johnson JM, Foerster JM, Hirsch CN, et al (2014) QTL mapping and phenotypic variation for root architectural traits in maize (*Zea mays* L.). Theoretical and Applied Genetics, 127, 2293-2311.

Cai H, Chen F, Mi G, Zhang F, Maurer HP, et al (2012) Mapping QTLs for root system architecture of maize (*Zea mays* L.) in the field at different developmental stages. Theoretical and Applied Genetics,125, 1313-1324.

Hund A, Fracheboud Y, Soldati A, Frascaroli E, Salvi S, Stamp P (2004) QTL controlling root and shoot traits of maize seedlings under cold stress. Theoretical and Applied Genetics 109, 618-629.

Landi P, Sanguineti MC, Darrah LL, Giuliani MM, Salvi S, Conti S, Tuberosa R (2002) Detection of QTLs for vertical root pulling resistance in maize and overlap with QTLs for root traits in hydroponics and for grain yield under different water regimes. Maydica 47:233–243.

Liu JC, Li JS, Chen FJ, Zhang FS, Ren TH, Zhuang ZJ and Mi GH (2008) Mapping QTLs for root traits under different nitrate levels at the seedling stage in maize (*Zea mays* L.). Plant and Soil 305:253–265

Liu J, Cai H, Chu Q, Chen X, Chen F, Yuan L et al (2011) Genetic analysis of vertical root pulling resistance (VRPR) in maize using two genetic populations. Molecular breeding, 28, 463-474.

Messmer R (2006) The genetic dissection of key factors involved in the drought tolerance of tropical maize (*Zea mays* L.). Diss. ETH No. 16695., Zurich, Switzerland

Osman KA, Tang B, Wang Y, Chen J, Yu F et al (2013) Dynamic QTL analysis and candidate gene mapping for waterlogging tolerance at maize seedling stage. PLoS One. 2013;8: e79305.

Ruta N, Liedgens M, Fracheboud Y, Stamp P et al (2010) QTLs for the elongation of axile and lateral roots of maize in response to low water potential. Theoretical and Applied Genetics, 120(3), 621-631.

Taramino G, Sauer M, Stauffer JL, Multani D, Niu XM, Sakai H, Hochholdinger F (2007) The maize (*Zea mays* L.) RTCS gene encodes a LOB domain protein that is a key regulator of embryonic seminal and post-embryonic shoot-borne root initiation. Plant Journal 50, 649-659.

Trachsel S, Messmer R, Stamp P, Hund A (2009) Mapping of QTLs for lateral and axile root growth of tropical maize. Theoretical and Applied Genetics 119:1413–1424

Tuberosa R, Sanguineti MC, Landi P, Michela Giuliani M, Salvi S, Conti S (2002) Identification of QTLs for root characteristics in maize grown in hydroponics and analysis of their overlap with QTLs for grain yield in the field at two water regimes. Plant Molecular Biology 48, 697-712.

Woll K, Borsuk LA, Stransky H, Nettleton D, Schnable PS, Hochholdinger F (2005) Isolation, characterization, and pericycle-specific transcriptome analyses of the novel maize lateral and seminal root initiation mutant rum1. Plant Physiology 139, 1255-1267.

Zhu J, Kaeppler SM, Lynch JP (2005) Mapping of QTL for lateral root branching and length in maize (*Zea mays* L.) under differential phosphorus supply. Theoretical and Applied Genetics 111:688–695.

Zhu J, Mickelson SM, Kaeppler SM, Lynch JP (2006) Detection of quantitative trait loci for seminal root traits in maize (*Zea mays* L.) seedlings grown under differential phosphorus levels. Theoretical and Applied Genetics,113(1), 1-10.
Zurek PR, Topp CN, Benfey PN (2015) Quantitative trait locus mapping reveals regions of the maize genome controlling root system architecture. Plant Physiology 167, 1487-1496.

Supplementary Figure 1. Frequency distribution for the 18 root and shoot seedling traits analyzed in the B73 x Gaspé Flint introgression line (IL) population (75 IL lines) utilized in this study. Trait acronyms and phenotypic values of parental lines are provided in Table 1.

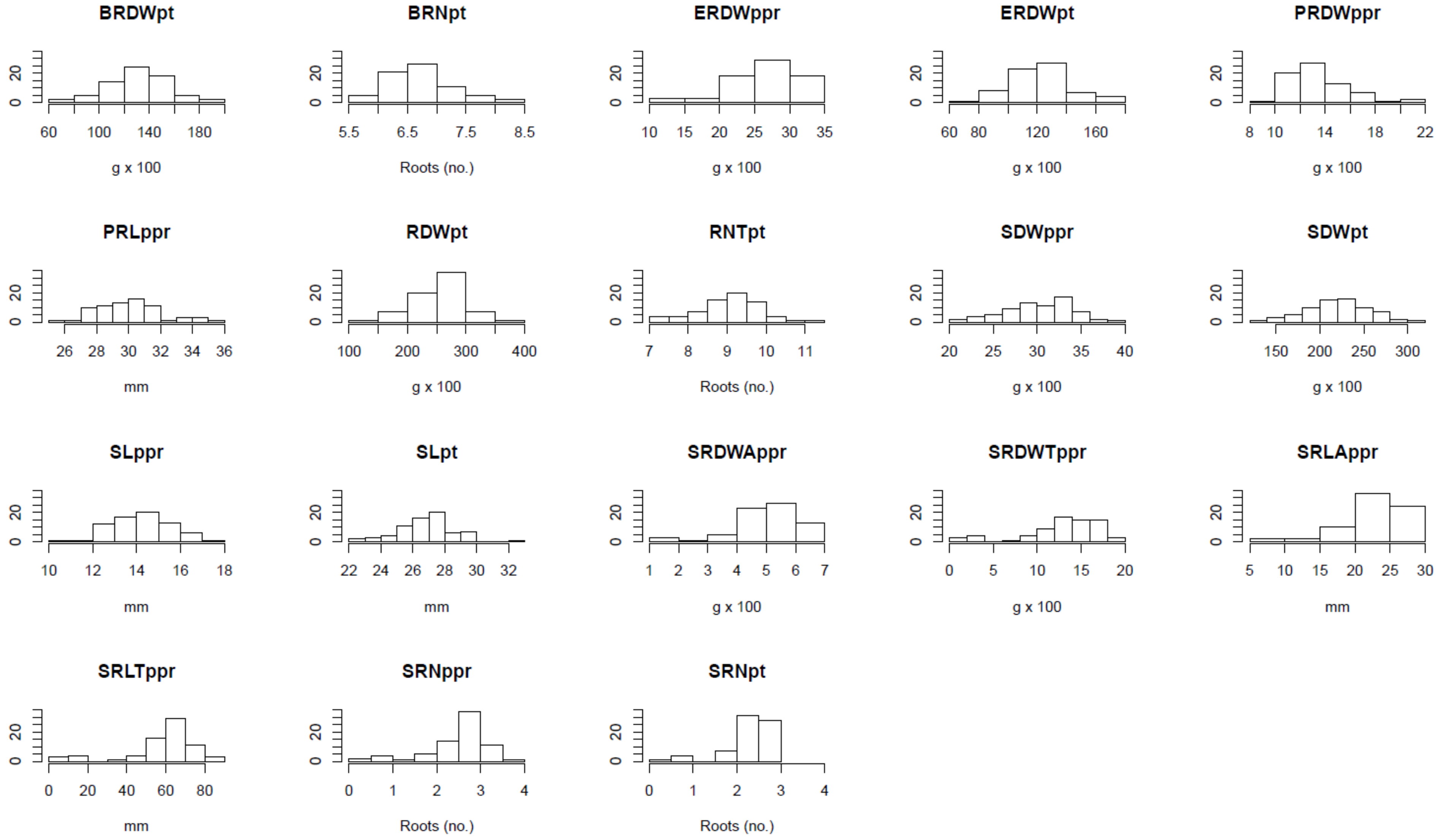

Supplement: Supplementary Data [file supp_erw011_supplementary_table_S1_S2_figure_S1.pdf]
